# A Common Neurodynamical Mechanism Could Mediate Externally Induced and Intrinsically Generated Transitions in Visual Awareness

**DOI:** 10.1371/journal.pone.0053833

**Published:** 2013-01-17

**Authors:** Theofanis I. Panagiotaropoulos, Vishal Kapoor, Nikos K. Logothetis, Gustavo Deco

**Affiliations:** 1 Department of Physiology of Cognitive Processes, Max-Planck-Institute for Biological Cybernetics, Tübingen, Germany; 2 Imaging Science and Biomedical Engineering, University of Manchester, Manchester, United Kingdom; 3 Department of Technology, Computational Neuroscience, Institució Catalana de Recerca i Estudis Avançats (ICREA), Universitat Pompeu Fabra, Barcelona, Spain; University of British Columbia, Canada

## Abstract

The neural correlates of conscious visual perception are commonly studied in paradigms of perceptual multistability that allow multiple perceptual interpretations during unchanged sensory stimulation. What is the source of this multistability in the content of perception? From a theoretical perspective, a fine balance between deterministic and stochastic forces has been suggested to underlie the spontaneous, intrinsically driven perceptual transitions observed during multistable perception. Deterministic forces are represented by adaptation of feature-selective neuronal populations encoding the competing percepts while stochastic forces are modeled as noise-driven processes. Here, we used a unified neuronal competition model to study the dynamics of adaptation and noise processes in binocular flash suppression (BFS), a form of externally induced perceptual suppression, and compare it with the dynamics of intrinsically driven alternations in binocular rivalry (BR). For the first time, we use electrophysiological, biologically relevant data to constrain a model of perceptual rivalry. Specifically, we show that the mean population discharge pattern of a perceptually modulated neuronal population detected in electrophysiological recordings in the lateral prefrontal cortex (LPFC) during BFS, constrains the dynamical range of externally induced perceptual transitions to a region around the bifurcation separating a noise-driven attractor regime from an adaptation-driven oscillatory regime. Most interestingly, the dynamical range of intrinsically driven perceptual transitions during BR is located in the noise-driven attractor regime, where it overlaps with BFS. Our results suggest that the neurodynamical mechanisms of externally induced and spontaneously generated perceptual alternations overlap in a narrow, noise-driven region just before a bifurcation where the system becomes adaptation-driven.

## Introduction

Multistable visual perception provides a unique window into the neural mechanisms that mediate visual consciousness. In such paradigms, two or more perceptual interpretations compete for access to awareness during periods of unchanged sensory stimulation. As a result, each time one of the possible interpretations is suppressed from conscious perception. Binocular rivalry (BR) and binocular flash suppression (BFS) represent two of the most extensively used paradigms of such perceptual suppression. In BR, two disparate visual patterns are continuously presented, usually through a stereoscope, in corresponding parts of the two retinas. This continuous ambiguity drives visual perception to fluctuate spontaneously between the two competing stimuli although retinal, sensory stimulation remains unchanged. Periods of stimulus dominance are followed by perceptual suppression in an unpredictable manner, characterized by stochastic temporal dynamics [1]–[2]. In BFS, after a brief period of monocular stimulation with a visual pattern, for example a face, a disparate visual stimulus is flashed to the contralateral eye [3]. This experimental manipulation results in the perceptual suppression of the first stimulus for at least one second. Thus, during this period the flashed stimulus becomes dominant by suppressing the contralateraly presented pattern. Obviously, although both BFS and BR induce perceptual suppression, in the former paradigm suppression is induced externally while in the latter it is spontaneously generated. Therefore, one intriguing question is whether the perceptual transitions in BFS and BR could be explained by a similar underlying dynamical mechanism.

Studies that combined BR or BFS with extracellular electrophysiological recordings in awake, behaving macaques suggest that in both paradigms two cortical neural representations of the stimuli compete for perceptual dominance [4]–[10]. In computational models of BR, these rivaling representations are conceptualized to involve two mutually inhibited neuronal populations, tuned to each competing visual pattern, that compete for activity dominance. The neuronal ensemble that is dominant or suppressed is believed to mediate stimulus awareness or suppression, respectively. In the past, different neurodynamical mechanisms have been suggested to mediate this competition involving adaptation, cross-inhibition and noise [11]–[29]. Most of these models describe two possible mechanisms that can achieve a switching in the dominance between those neuronal populations, as observed in BR. The first mechanism (“*adaptation-driven*” with cross-inhibition between the two competing pools) assumes that a slow adaptation process provokes a fatigue of the dominant activity and a decrease of cross-inhibition such that the suppressed population can take over the competition, becoming active and inactivating the first originally dominant population [12], [16], [18]–[24]. The role of adaptation in BR is well supported by empirical studies showing a gradual decrease in the strength of the dominant stimulus and an increase in the probability of a perceptual switch as a function of increasing dominance duration [3], [25].

A second possible mechanism that was first exposed by Moreno-Bote et al. [17] is known as “*noise-driven*” and assumes that the underlying neurodynamical system is bistable. In this scenario, noise is the main source provoking a transition between the two bistable states, by causing the jump over the barrier separating the two stable attractors of the system [17]–[18], [23]–[30]. In these models when noise is absent, alternations are impossible since the system relaxes indefinitely in one of the two attractors. The most important evidence for the role of noise in perceptual transitions is the stochastic temporal dynamics of the perceptual transitions observed during BR. Recent theoretical work suggests that both adaptation and noise operate in a fine balance to induce the stochastic properties of the perceptual alternations observed in BR [25]–[28].

In this study, we compare the dynamical range of BR and BFS. We constrain a single unifying stochastic neuronal competition model with neurophysiological data obtained from recordings in the lateral prefrontal cortex (LPFC) during BFS. By changing the level of adaptation, our model goes from a bistable regime, where transitions are noise-driven, to an oscillatory regime, where transitions are adaptation-driven. We then compare the neurodynamical range of BFS to the respective range of BR, constrained by known psychophysical parameters. Our results demonstrate the non-trivial fact that the working region of the system as constrained by BR (i.e. a noise-driven region at the edge of the bifurcation) overlaps significantly with the working region as constrained with the averaged neuronal discharge pattern observed in the LPFC during BFS.

## Results

**Figure 1:**
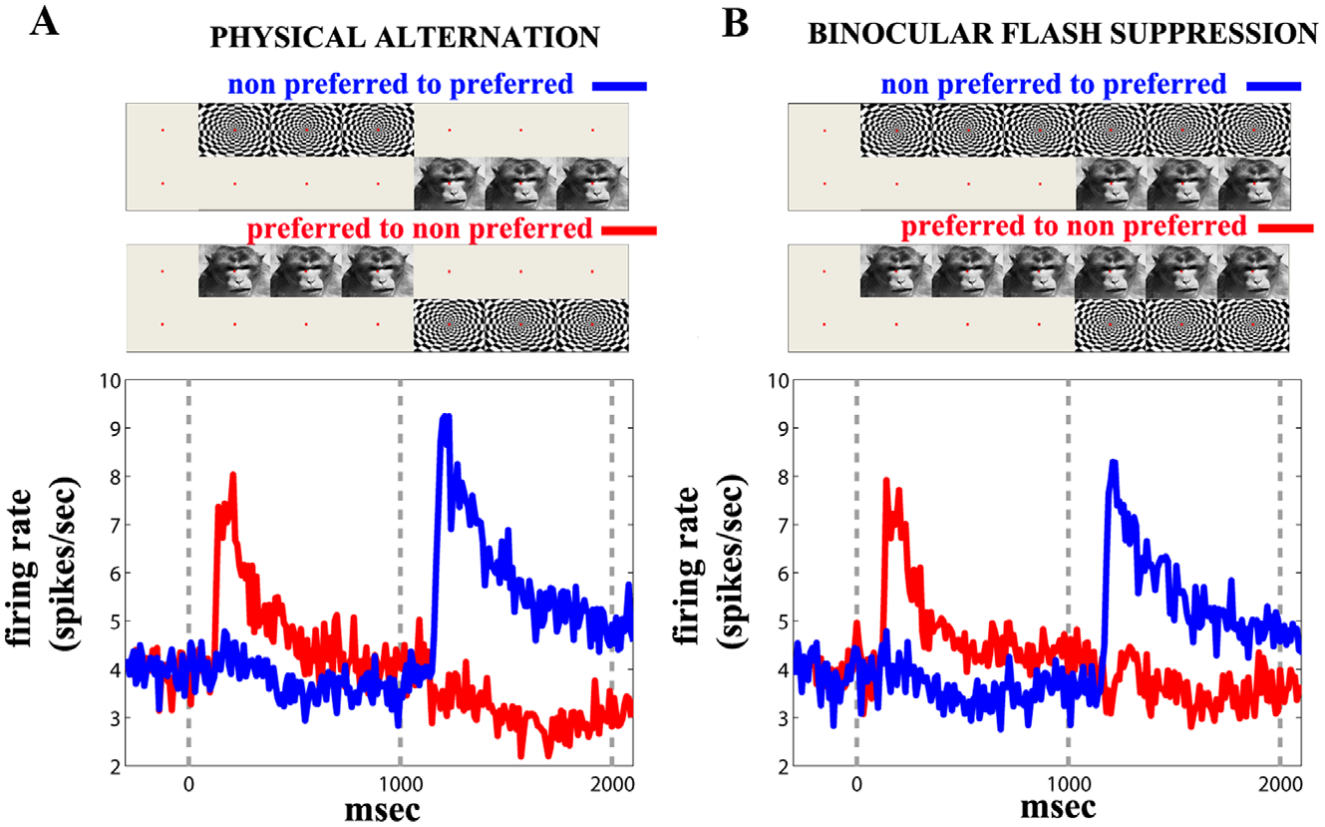
Mean population response in the LPFC during physical alternation and BFS (adapted and modified from [**7**]). (A) Mean population activity averaged across units showing significant sensory modulation during monocular physical alternation between disparate stimuli. Blue trace shows the mean activity when visual stimulation starts from a non-preferred stimulus followed by switching to a preferred visual pattern. Red curve depicts the mean activity when visual stimulation starts with a preferred pattern followed by switching to a non-preferred stimulus in the contralateral eye. (B) Same a (A) for BFS. Perceptual dominance of a preferred stimulus (blue, *t* = 1001–2000 msec) results in increased spiking activity similar to that observed during physical alternation of the same stimuli. When the same stimulus is perceptually suppressed (red, *t* = 1001–2000) the mean population activity remains suppressed until the end of the trial.

We recently found that feature-selective populations in the LPFC follow the phenomenal perception of a preferred stimulus [7] (Figure 1). Specifically, a pool of feature-selective neurons showing reliable stimulus preference during monocular stimulation (i.e. monocular physical alternation of two disparate stimuli), retains its preference during BFS (i.e. when the preferred stimulus is perceived during visual competition). As depicted in Figure 1A, following monocular stimulus alternation from a non preferred to a preferred pattern (blue curve), the mean neuronal discharge activity rapidly increased and then slowly adapted but remained visually modulated, much above the baseline activity elicited by simple fixation. In trials where a transition to a non preferred visual pattern followed the initial, monocular presentation of the contralateral eye with a preferred stimulus, the averaged firing rate decreased (red curve). The mean discharge activity pattern was found to be almost identical during BFS (Figure 1B). More specifically, the perceptual dominance of a preferred pattern, resulted in a significant increase of the mean population firing rate, very similar to the increase observed during physical alternation. In a similar fashion, a pattern identical to the physical alternation was obtained when a preferred stimulus was perceptually suppressed. For a more detailed description of the neurophysiological data see [7].

**Figure 2:**
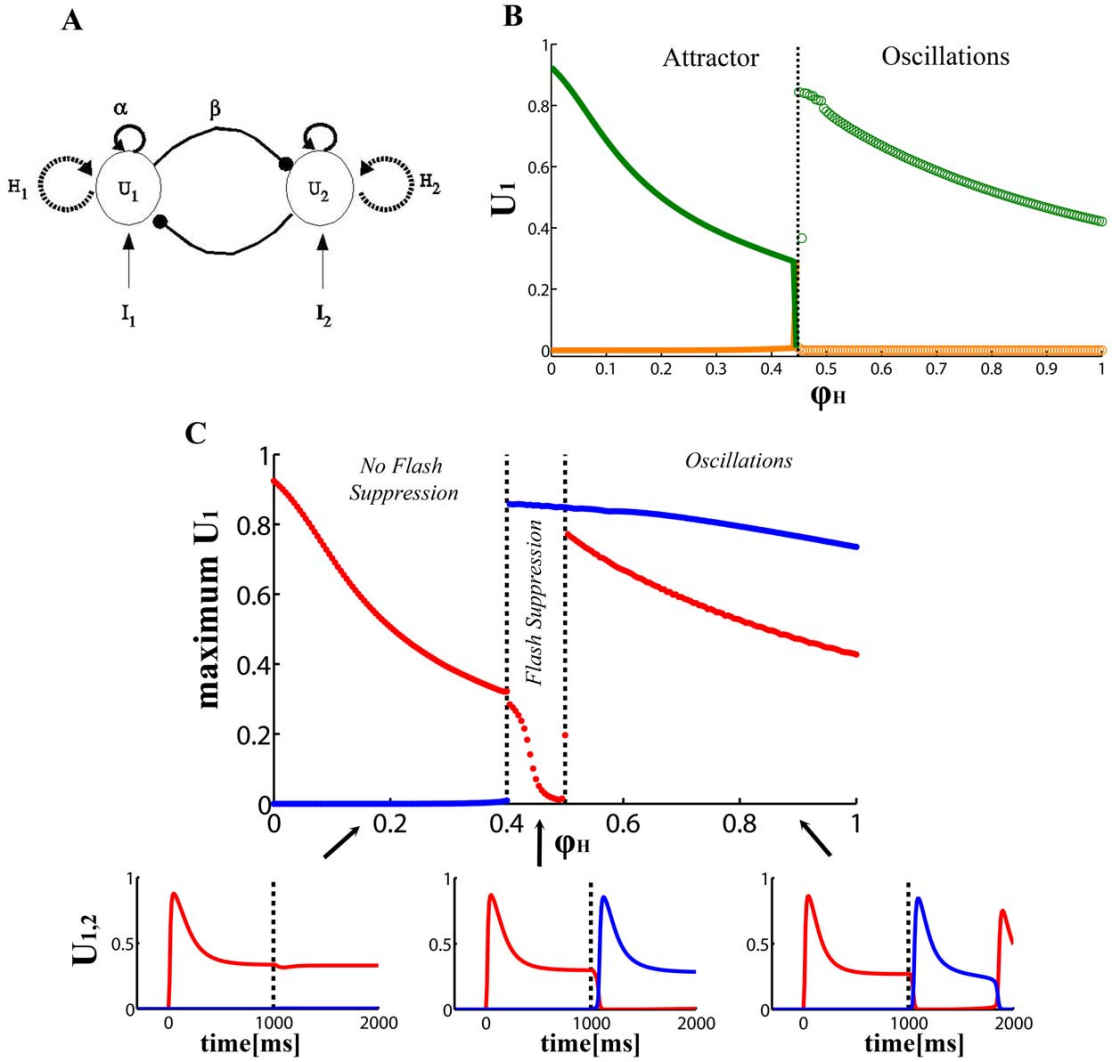
Bifurcation analysis of BFS. (A) Neuronal Competition Network adaptation-LC. The population rate activity is denoted by U_i_ on each neuronal population i. The external visual input on each eye (population) *i* is denoted by *I_i_*. Mutual inhibitory connections *β* are represented by filled circles, and recurrent excitatory connections *α* by arrows. The recurrent dashed arrows on each population symbolize a slow adaptation process (rate frequency adaptation), where the adaptation variable is denoted by 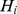 on each population *i*. (B) Bifurcation diagram of the noise free mode as a function of the strength 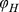 of the adaptation process. A bifurcation from a bistable (“Attractor”) regime to an oscillatory (“Oscillations”) regime is observed at 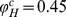. Left of the bifurcation, a double branched bistable region (solid lines) emerges. The upper (green) and lower (orange) branches correspond to the high and low activity of the dominant and suppressed population, respectively. Right of the bifurcation, an oscillatory region emerges. In this region, the maximum and the minimum of the populations’ activity during rivalry periodic oscillations (green and orange circles) are shown. (C) Bifurcation analysis of BFS. Upper panel: Flash suppression dynamical range of the competition neuronal model for the noise free case. Flash suppression is characterized by plotting the maximum value of the populations’ activity (*U_1_* in red and *U_2_* in blue) during the last second of the simulations (see details in the text). Flash suppression corresponds to the regions where only the second flashed population *U_2_* shows high activity and the first stimulated population *U_1_* is suppressed. Lower panel: Temporal evolution of the populations’ firing rate activity *U_1_* (red) and *U_2_* (blue) for different levels of adaptation corresponding to the three different regions (pointed by the black arrows) observed in the upper panel. Neurophysiological single cell recordings in monkey LPFC are only consistent with the type of simulated neuronal behavior observed in the region labeled “Flash Suppression”.

These results show that neuronal discharges in the LPFC reliably reflect the outcome of visual competition during BFS (i.e. the content of visual consciousness), for at least one second of rivalrous stimulation. Here we constructed a realistic competition model (Figure 2A) and simulated the experimental procedure of BFS to discern the dynamical range in which a discharge pattern similar to the pattern previously recorded in the LPFC during BFS is observed. We focused our investigation on the two parameters previously shown to regulate the dynamical regimes of rivalrous visual stimulation, the strength of the adaptation process 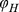 and the level of noise *σ* [27]. This unified model, evaluating simultaneously the role of adaptation and noise could elucidate the relative contribution of each factor in perceptual transitions.

First, we performed a bifurcation analysis of the dynamical system given by equations 2 and 4, for the noise free case, i.e. for *σ* = 0 and plotted the stable stationary states of the model as a function of adaptation 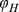
in Figure 2B. The bistable (“Attractor”) range is the double-branched region before the bifurcation point at 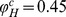. The top (green) branch corresponds to the high firing rate activity of the dominant population, whereas the bottom (orange) branch corresponds to the low firing rate activity of the suppressed population. The bistable states arise in a symmetric way just by interchanging the label of the dominant/suppressed populations. After the bifurcation the bistability disappears and an oscillatory (“Oscillations”) region emerges. In this noise free case, if the working point of the system is in the region before the bifurcation (at 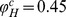), the system cannot escape one of the two stable states and consequently cannot show activity alternations and thus rivalry. In this regime, alternations can happen only due to the non-deterministic force of noise. On the other hand, if the dynamical working point is in the oscillatory region after the bifurcation, then the system will oscillate (exhibiting activity transitions) at a periodic rhythm which depends on the strength of 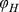. In that case the system will show transitions of activity but the temporal dynamics of these transitions will be periodic and not stochastic, as in BR. Moreover, in this regime, adaptation is the critical factor that drives perceptual transitions.

Next, we investigated, for the noise free case, the dynamical range where BFS emerged. For this case, we performed a simulation that emulated exactly the BFS design utilized for the electrophysiological recordings in the macaque LPFC. More specifically, we started the simulation with a period of 300 ms without retinal stimulation, i.e. with *I_1_* =  *I_2_* = 0. Following this period, a monocular stimulus was presented (population *U_1_*) for 1000 ms, i.e. for this period we set *I_1_* = 0.5 and *I_2_* = 0. During a final period of 1000 ms, the second stimulus was flashed to the contralateral eye (population *U_2_*), i.e. *I_1_* =  *I_2_* = 0.5. For characterizing the dynamical regimes where the model showed flash suppression as in the neurophysiological experiments, we plotted in Figure 2C the maximum value of the populations’ activity (*U_1_* in red and *U_2_* in blue) during the last second of the simulations (i.e. after the flash of the second stimulus). Only a narrow region around the bifurcation point 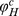 is consistent with the mean population discharge pattern in the LPFC during BFS. In the first region (labeled “*No Flash Suppression*”, Figure 2C), the population *U_2_*, corresponding to the second flashed stimulus, is not able to win the competition, because the competing population *U_1_* is not sufficiently adapted (Figure 2C, lower left panel). In the second region (labeled “*Flash Suppression*”), the level of adaptation is adequate to allow population *U_2_* to win the competition during the whole last second (Figure 2C, lower middle panel). In the third region (labeled “*Oscillations*”), the level of adaptation is too large, and consequently, the population *U_2_* wins the competition, but the suppressed stimulus is able to win the competition again before the end of the trial (i.e. in less than 1 second) (Figure 2C, lower right panel). The mean discharge pattern in the LPFC presented in Figure 1 shows that the phenomenon of BFS correlates with the type of responses observed in the region labeled “*Flash Suppression*” (depicted in the lower middle panel of Figure 2C). In the bifurcation diagram, the model matches the neurophysiological recordings in a region around the bifurcation, for 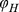 values between 0.4 and 0.5.

**Figure 3:**
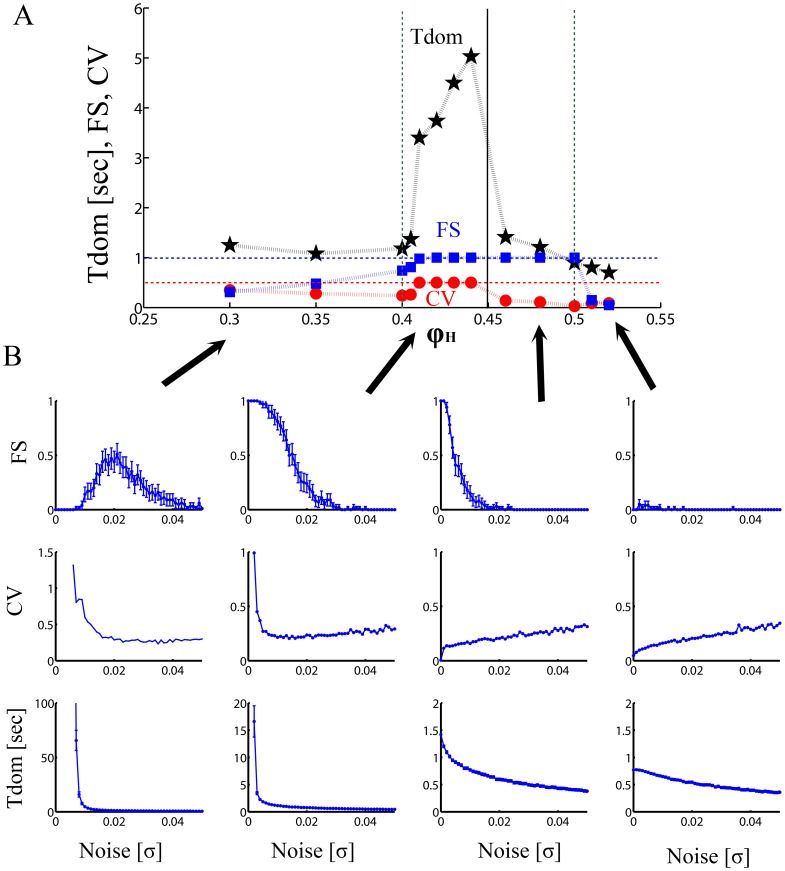
Effect of noise on BFS. (A) Flash suppression (*FS*), Binocular rivalry dominance time (*T_dom_*), and the coefficient of variation (*CV*) as a function of the adaptation level 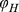, and with a level of noise *σ* optimized for each point such that the *FS* index is maximal and the *CV* is as near to 0.5 as possible. The black stars indicates the value of *T_dom_*, the blue squares *FS*, and the red dots the corresponding *CV*. There is only one dynamical region where the model is consistent with the experimental constraints, namely a narrow region just before the bifurcation 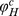. For this region the level of adaptation 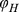 is such that an optimal level of noise exists for which all neurophysiological (BFS) and psychophysical (BR) constraints can be met. (B) Model simulations, for different fixed levels of adaptation, showing the dependence of these measurements (FS, *T_dom_*, CV) on the noise. In this figure four different values of 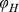, corresponding to different regions in the bifurcation diagram, are shown.

In order to further constrain the dynamical range of the neuronal competition model, we considered the influence of the noise level on the phenomenon of BFS, and on the typical psychophysical dynamics of the alternation under BR conditions, namely the mean dominance time (*T_dom_*), and the coefficient of variation (*CV*, defined as the ratio between the standard deviation and mean of the transition times). For describing flash suppression (blue squares in Figure 3A), we detected the number of trials *M* (each one with the flash suppression scheme described above, but now with noise) that during the last second (after the second stimulus is flashed) showed the flash suppression effect as evidenced in the single cell recordings in the monkey LPFC, i.e. only the population associated with the flashed stimulus shows a high activation peak. We performed 100 trials and defined the flash suppression performance variable *FS* = *M/100.*

We simulated for different fixed levels of adaptation, the dependence of these measurements on noise (Figure 3). The known experimental constraints demand that *FS* be near 1, *CV* near *0.5* and *T_dom_* between approximately 1 and 10 seconds [5]–[9], [31]–[36]. In our psychophysical measurements (in one macaque) for the stimuli used during the electrophysiological recordings the *T_dom_* of the flashed stimulus (thus during rivalrous stimulation) was 2.55±0.35 (mean±S.E.M) and the *CV* = 0.46 (see also supplementary results in [7] ). Furthermore, more than 60% of the observations yielded a dominance duration above 2 seconds.

Figure 3A depicts that there is only one dynamical region where all the experimental constraints (analytically described in Figure 3B) are satisfied, namely a narrow region just before the bifurcation. The narrow region after the bifurcation 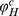 also shows a good behavior in agreement with the BFS neurophysiological data, but the level of *CV* (red circles) and *T_dom_* (black stars) are inconsistent with BFS. Although *CV* and *T_dom_* measures within the reported range of previous studies can be obtained in various areas around the bifurcation (also shown in [27]), in these areas the *FS* parameter is not realistic based on our observations. Therefore, the dynamical range of BFS and BR is more likely to overlap in a narrow, noise-driven region before the bifurcation (for 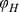 values between 0.4 and 0.45) separating the noise-driven from the oscillatory-driven regime.

**Figure 4:**
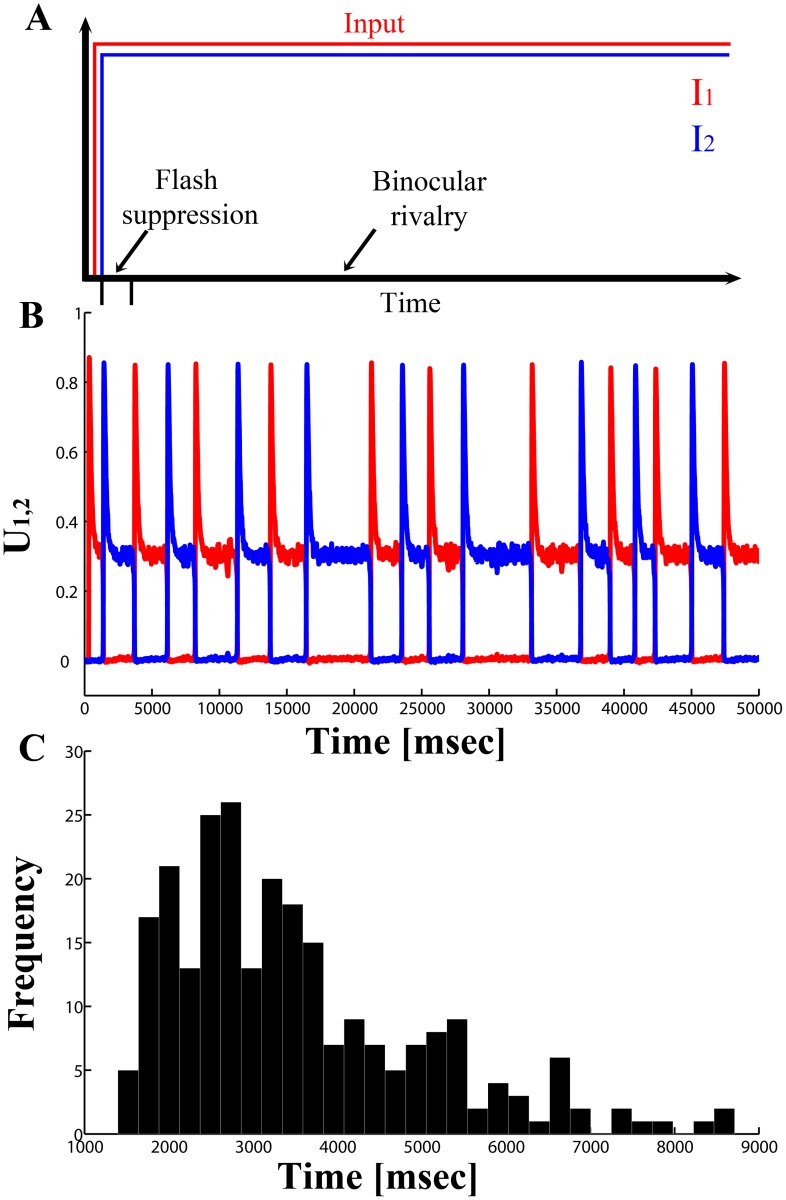
Neuronal dynamics during BFS and BR for optimal noise levels. (A) Schematic depiction of the simulated experiment performed for BFS and BR. Input *I_1_* is followed by input *I_2_*, simulating flash suppression. Then, both inputs continue to be present, resulting in binocular rivalry. (B) Firing rate activity of the populations in the neuronal competition model for a fixed value of 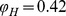 in the good dynamical range. The figure shows a single trial, that starts with flash suppression, but then the two visual inputs keep stimulating both populations, so that binocular rivalry emerges. During the first 2300 ms, flash suppression activity, as observed in monkey prefrontal neurons, appears. After that, we observe the typical irregular alternations between both populations (and therefore between both percepts). (C) Distribution of the alternation times. The model simulation yields a gamma-like distribution with mean dominance time *T_dom_* = 3.9 seconds and a *CV* = 0.5.

Finally, we studied the system under BR conditions, by setting *I_1_* =  *I_2_* = 0.5 for 100 seconds in order to have a sufficient number of perceptual transitions (Figure 4A). The typical behavior of the neuronal system for a value of 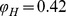 in the good, noise driven, dynamical range is characterized by BFS (Figure 4B, first activity transition) but also by activity transitions with temporal dynamics resembling those of BR when we simulate rivalrous stimulation (Figure 4B, 4C). The model simulation yields a gamma-like distribution with mean dominance time *T_dom_* = 3.9 seconds and a *CV* = 0.5 consistent with the known behavioral observations.

Finally, we note that contrary to previous work [27], we stress here the necessity of optimizing the noise level independently for each working point (see Figure 3C), in order to consider properly the large effect of fluctuations especially at the edge of bifurcations. Furthermore, we show that by doing this, we can constrain the region consistent with the experimental data to a narrow region at the edge of the bifurcation but still in the noise driven region, changing and specifying therefore even more the mechanisms underlying bistable perception suggested by previous works [17], [25]–[27].

## Discussion

BFS is nowadays very well phenomenologically characterized by quantitative descriptions of its associated behavior and underlying neurophysiology [3], [6]–[10]. How can we fuse these two levels of information for gaining a unifying and more complete explanation of the underlying computation? To understand the neuronal mechanisms underlying BFS, we need to consider and analyze the neuronal and behavioral data from a system dynamics perspective that enables us to study explicitly that dynamics. The description should be simple enough to enable us to infer by abstraction the first principles or computational correlates of the brain function(s) under study. In this sense, we are faced with an inverse problem: We have to extract the free parameters of a system that cannot be measured directly (e.g., the connectivity between the thousands of neurons making up any plausible sub network) but that can be inferred by (a) studying the dynamical capabilities of the system and (b) looking for regions within a parameter space that generate an emergent behavior consistent with the experimentally measured observations.

Neurophysiological evidence gives rise to the assumption that a cortical area is capable of representing a set of alternative hypotheses encoded in the activities of disparate cell assemblies. Representations of different conflicting hypotheses inside each area compete with each other for activity and for being represented [37]. In parallel to this competition-centered view, a cooperation-centered picture of brain operation has been formulated, where a given hypothesis representation finds its neural correlate in assemblies of co-activated neurons [38]. The concept of neural assemblies has been formalized in the framework of statistical physics [39]–[42] where assemblies of co-activated neurons form attractors in the phase space of the recurrent neural dynamics (patterns of co-activation can represent fixed points to which the dynamical system evolves). To model brain dynamics we can use therefore an attractor network model [43]. This type of attractor network of neurons is a dynamical system that in general has the tendency to settle in stationary states, fixed points called “attractors”, typically characterized by a stable pattern of firing activity. External or even intrinsic noise that appears in the form of finite size effects could provoke destabilization of an attractor inducing therefore transitions between different stable attractors. The dynamics of the network can be described by coupling the dynamical equations describing each neuron and the synaptic variables associated with their mutual coupling. In studying such a system there is a question as to how to set the parameters which are not biologically constrained by experimentally determined values. The standard trick is to simplify the dynamics via the mean-field approach [44] and to analyze the bifurcation diagrams of the dynamics in order to solve the above mentioned inverse problem. A bifurcation diagram shows the possible dynamical states of the system as a function of the model parameters. This enables a posteriori selection of the parameter region which in the bifurcation diagram shows the emergent behavior of interest (e.g. BFS) and in this way we can understand the working point and the particular computation underlying the physiological and behavioral data.

Consequently, here we used a mean-field (rate-like) reduced model and performed explicitly the solution of the inverse problem by detecting in which region of the bifurcation diagram the neuronal and behavioral correlates of BFS are explained. Our mechanistic simulations demonstrate that the mean population discharge pattern observed during externally induced perceptual alternations (BFS) in the LPFC can only be obtained in a narrow region around the bifurcation separating a noise-driven, attractor regime from an adaptation-driven, oscillatory regime. Therefore, our results suggest that neither noise nor adaptation forces alone have a primary, crucial role in externally induced (BFS) perceptual switches. The population discharge pattern observed in the LPFC during BFS could be the result of either adaptation or noise alone. Assuming that we start with BFS and then we let the stimuli rival in BR there are two possibilities. If the system operates in the attractor regime from the very beginning (i.e. if the BFS alternation is due to a causal influence of noise), the same level of noise would subsequently lead to BR with stochastic temporal dynamics similar to the ones observed experimentally (Figure 4B, C). In that case, the system could operate in the attractor regime for both BFS and BR, indicating that both paradigms share similar neurodynamical mechanisms. However, the fact that BFS can also be obtained in a narrow region immediately after the bifurcation, leaves open the possibility that BFS could be the result of a mainly adaptation-driven oscillatory mechanism. Since this region gives extremely low *CV* and *T_dom_* values (Figure 3A) it is possible that in an experimental design where BFS occurs first and then is followed by BR, there is a switch in the dynamical mechanisms themselves (i.e. initially BFS could be mediated by adaptation-driven oscillatory mechanisms but subsequently, noise-driven attractor mechanisms could take over during BR).

The predictions of the model presented here are in agreement with the psychophysical observations described in the original paper on the phenomenon of BFS [3]. More specifically, Wolfe showed that when the duration of the initial, adapting stimulus is less than 200msec (and thus when spiking activity in the LPFC is still at its peak and adaptation has not yet been manifested) then the magnitude of perceptual suppression and thus the probability of a perceptual transition, is low. At the same time this lower time limit does not necessarily mean that adaptation to the initial pattern is a sufficient factor for perceptual suppression since a long monocular stimulus followed by an ipsilateral flash does not produce suppression of the flashed pattern which can be easily detected. Rather it seems that the time scale necessary for adaptation to occur is increasing the probability of a transition and adaptation is only one of the necessary factors (the other obviously being visual competition) that contribute to a perceptual switch.

Furthermore, recent psychophysical work showed that percept strength gradually decreases during dominance, due to adaptation [25]. However, psychophysical and theoretical studies have previously suggested that adaptation is not sufficient to explain the perceptual alternations without the influence of stochastic fluctuations [17], [23], [25]–[29], [31]–[33], [35]–[36]. Indeed, more recently, a theoretical study using only psychophysical experimental data as constraints, showed that the mechanism producing perceptual alternations operates near the boundary between adaptation and noise driven transitions [27]. To our knowledge, our study is the first to demonstrate that the neuronal correlates of an ambiguous perception paradigm (here BFS), in a cortical area where neuronal discharges follow perception (LPFC), are indeed found to occur within this boundary.

The mechanism suggested here, and validated by the neurophysiological data, implies that adaptation progressively decreases the stability of the dominant state (similar to the mechanism suggested in [27] ), but just until the point that the state gets unstable. The adaptation is not able to provoke a transition, but an infinitesimal increase of it would do so. In our case, noise drives the alternation even before the dominant state get unstable. Furthermore, only this dynamical range is able to explain the high irregularity of activity alternations evidenced by a high value of *CV* = 0.5, while at the same time being able to catch the perception of a flashed stimulus. We suggest that the system has a balanced working point at the edge of the bifurcation between noise and adaptation, which is optimal in the sense of maximal sensitivity. This results in BFS which is optimal in the sense that a newly flashed stimulus is immediately perceived for at least one second. Future efforts should concentrate on identifying reliable single unit correlates of intrinsic perceptual transitions (e.g. BR) to further confirm the validity of this dynamical mechanism.

Finally, we point out that the neuronal responses used to constrain our model were recorded from the macaque LPFC, a cortical area shown to reflect reliably the content of conscious visual perception [7]. The human PFC is also involved in the temporal dynamics of transitions between different representations during ambiguous stimulation as evidenced in patients with prefrontal cortex lesions [45]–[47]. Taking this evidence into account, the results and the interpretation presented here assume that the dynamics of competition at this stage of cortical processing, acting between explicit representations of neuronal activity, is a critical factor for perceptual transitions. The source of noise could be attributed either to the local dynamics in this area or to fluctuations in the input, inherited from earlier areas in the visual cortical hierarchy, and affecting the neuronal dynamics in the LPFC.

## Materials and Methods

### Ethics Statement

All animal procedures were approved by the local authorities (Regierungspräsidium Tuebingen) and were in full compliance with the guidelines of the European Community (EUVD 86/609/EEC) for the care and use of laboratory animals. See also [7].

### Electrophysiological Recordings and Theoretical Framework

Materials and methods as well as a detailed analysis of the electrophysiological recordings can be found in [7]. We consider here a common and standard neuronal competition model called the “Adaptation-LC” model [16]. The architecture of the model is schematized in Figure 2A. The model consists of two populations of neurons, each one being sensitive to the visual pattern presented on each of the two eyes, respectively. We denote the input to population *i* by 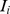. In the absence of stimulation on one eye *i* (as for example in the first period of a flash suppression experiment), the value of 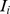 is equal to zero.

This type of neuronal circuit is able to sustain dynamical regimes of multi-stability. In these multi-stable regimes, fluctuations are needed to drive the transitions. If the neuronal populations are comprised of large numbers of spiking neurons, fluctuations arise naturally through noisy input and/or disorder in the collective behavior of the network. The dynamical behavior of such large-scale networks can be captured in a system of nonlinear coupled differential equations that describes the evolution of the average firing rate of each population (mean field reduction, see [44]). In this case, a fluctuation term must be added to drive the transitions. Such a minimal reduced network consists of two distinct populations of neurons. Neurons within a specific population interact via strong recurrent excitation with weight *α*. Neurons in one population are mutually coupled to all other neurons in the other population in an inhibitory fashion with a weight *β*.

The temporal dynamics of the firing rates of the neuronal populations can be qualitatively captured via a system of first order differential equations of the Wilson-Cowan-type [44]. In this paper, we use Wilson-Cowan rate model, because it is simple to analyze, but is biologically realistic in the sense that it corresponds to the mean-field reduction of a complex network of spiking integrate-and-fire neurons. Simulations of such networks of spiking neurons are computationally expensive, which makes them rather unsuitable for systematic parameter explorations. The standard trick to solve this problem is to simplify the dynamics via the “mean field” approach and to analyze there the bifurcation diagrams of the dynamics. The essence of the mean-field approximation is to simplify the integrate-and-fire equations by replacing after the diffusion approximation, the sums of the synaptic components by the average DC component and a fluctuation term. The stationary dynamics of each population can be described by the “population transfer function”, which provides the average population rate as a function of the average input current.

For the BR network shown in Figure 2A the firing rate equations are the following:
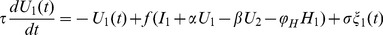
(1)

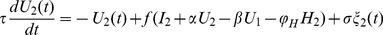
(2)

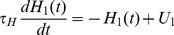
(3)

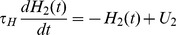
(4)where 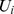 denotes the firing rate of population *i*, 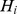 its slow adaptation variable and 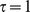
msec. The nonlinear transfer response function *f*(·) is the sigmoidal function:



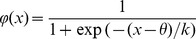
(5)Throughout this study *α* = 0, *β* = 1, *τ_H_* = 50, *τ* = 1, *k* = 0.1, *θ* = 0.4, and *I_i_* = 0.5 or *I_i_* = 0 when the stimulus image is presented to the eye *i* or not, respectively. In this work we manipulate two free parameters, namely the level of adaptation, regulated by 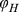 and the level of fluctuations, regulated by *σ* (the standard deviation of the noise).

Here, fluctuations are modeled via an additive Gaussian noise term denoted by 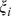. Here 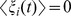 and 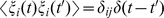, where the brackets 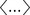 denote the average over stochastic random variables. This noise term represents finite-size effects that arise due to the finite number, *N*, of neurons in the populations. We note that there are two sources of noise in such spiking networks: the randomly arriving external Poissonian spike trains and the fluctuations due to the finite size of the network. Here, we concentrate on finite-size effects due to the fact that the populations are described by a finite number, *N*, of neurons. In the mean-field framework “incoherent” fluctuations due to quenched randomness in the neurons’ connectivity and/or due to external input are already taken into account in the variance, and “coherent” fluctuations give rise to new phenomena [48], [49]. In fact, the estimate of 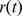, probability of emitting a spike per unit time in the infinite network, is then a stochastic process 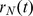, well described in the limit of large *N* by 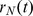
≃ 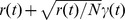, where 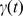 is Gaussian white noise with zero mean and unit variance. The stochastic equations were solved using Euler’s forward method.

For the noise free case, we study the dynamical range where BFS emerges. For this case, we perform a simulation that emulates exactly the BFS design utilized for the electrophysiological recordings in the macaque LPFC. More specifically, we start the simulation with a period of 300 ms without retinal stimulation, i.e. with *I_1_* =  *I_2_* = 0. Following this period, a monocular stimulus is presented (population *U_1_*) for 1000 ms, i.e. for this period we set *I_1_* = 0.5 and *I_2_* = 0. During a final period of 1000 ms, the second stimulus is flashed to the contralateral eye (population *U_2_*), i.e. *I_1_* =  *I_2_* = 0.5. For describing the effect of noise on BFS, we detect the number of trials *M* (each one with the flash suppression scheme described above, but now with noise) that during the last second (after the second stimulus is flashed) show the BFS effect as evidenced in the single cell recordings in the macaque LPFC, i.e. only the population associated with the flashed stimulus shows a high activation peak. We performed 100 trials and defined the BFS performance variable *FS* = *M/100*. Furthermore, we study the system under BR conditions, i.e. we set *I_1_* =  *I_2_* = 0.5 during 100 seconds in order to have enough alternations.
